# Epithelia-derived wingless regulates dendrite directional growth of *drosophila* ddaE neuron through the Fz-Fmi-Dsh-Rac1 pathway

**DOI:** 10.1186/s13041-016-0228-0

**Published:** 2016-04-29

**Authors:** Xiaoting Li, Yan Wang, Huan Wang, Tongtong Liu, Jing Guo, Wei Yi, Yan Li

**Affiliations:** State Key Laboratory of Brain and Cognitive Science, Institute of Biophysics, Chinese Academy of Sciences, Beijing, 100101 China; University of Chinese Academy of Sciences, Beijing, 100049 China; Beijing Institutes of Life Science, Chinese Academy of Sciences, Beijing, 100101 China

**Keywords:** Epithelia-derived Wingless, Dendrite directional growth, Frizzled, Flamingo, non-canonical Wnt pathway

## Abstract

**Background:**

Proper dendrite patterning is critical for the receiving and processing of information in the nervous system. Cell-autonomous molecules have been extensively studied in dendrite morphogenesis; however, the regulatory mechanisms of environmental factors in dendrite growth remain to be elucidated.

**Results:**

By evaluating the angle between two primary dendrites (PD-Angle), we found that the directional growth of the primary dendrites of a *Drosophila* periphery sensory neuron ddaE is regulated by the morphogen molecule Wingless (Wg). During the early stage of dendrite growth, Wg is expressed in a group of epithelial cells posteriorly adjacent to ddaE. When Wg expression is reduced or shifted anteriorly, the PD-Angle is markedly decreased. Furthermore, Wg receptor Frizzled functions together with Flamingo and Dishevelled in transducing the Wg signal into ddaE neuron, and the downstream signal is mediated by non-canonical Wnt pathway through Rac1.

**Conclusions:**

In conclusion, we reveal that epithelia-derived Wg plays a repulsive role in regulating the directional growth of dendrites through the non-canonical Wnt pathway. Thus, our findings provide strong *in vivo* evidence on how environmental signals serve as spatial cues for dendrite patterning.

**Electronic supplementary material:**

The online version of this article (doi:10.1186/s13041-016-0228-0) contains supplementary material, which is available to authorized users.

## Background

Dendrites receive and process most of the information from external stimuli and input neurons; therefore, it is essential for dendrites to develop an elaborate arborization pattern. Both cell-intrinsic and extrinsic factors play instructive roles in regulating dendrite development [1]. A number of intrinsic factors, such as transcriptional factors, organelles, and regulators of cytoskeleton, provide internal forces for dendrite growth and branching [2, 3]. On the other hand, extrinsic factors from the environment serve as spatial cues to guide dendrites to their targeting areas and ensure the extension direction and branching pattern. In mammalian cortical plate, several extrinsic factors, including Semaphorin3A (Sema3A), Slit, Brain-derived neurotrophic factor (BDNF), and Notch, are coordinated to direct the growth of apical dendrites towards the pial surface, and thus control the dendrite patterning in pyramidal cells [4–7]. In *C. elegans*, two epithelial adhesion molecules, SAX-7 and MNR, and a neuronal receptor, DMA-1, form a tripartite ligand-receptor complex to provide spatial information for dendrite branching in the PVD neurons [8, 9]. Similarly, in *Drosophila*, several extrinsic factors have been reported to guide the dendrites to their targeting sites, including Sema1A, Netrin and Slit [10, 11]. These earlier studies indicated that environmental cues play essential roles in regulating the directional growth of dendrites in *Drosophila*.

*Drosophila* dendritic arborization (da) neurons extend their dendrites in a 2D plane at the basal surface of the epidermis in contact with extracellular matrix (ECM) [12, 13]. These da neurons are classified into four classes, according to their stereotyped dendritic fields and branching complexities, and have been used as an ideal model system for studying dendrite morphogenesis [3]. It has been reported that extrinsic factors originating from the ECM and epithelium adjacent to da neurons regulate the tiling, scaling and self-avoidance processes of class IV da neurons [12–16]. These factors play essential roles in establishing and maintaining the radial dendrite pattern of class IV neurons, therefore ensuring that the neurons cover the whole body wall of larvae. Different from class IV neurons, class I da neurons possess comb-like dendritic arborizations, with their primary and secondary dendrites extending along the dorsal-ventral (DV) and the anterior-posterior (AP) directions, respectively. A study from T. Uemura’s lab indicated that Ten-m, a homophilic cell adhesion molecule of the Teneurin family, is highly expressed in the class I neuron ddaE, where it was found to regulate directional control of dendritic sprouting and extension through homodimer interactions with epidermal Ten-m molecules. The secondary dendrites of the ddaE neuron respond to the Ten-m gradient along the AP direction in epidermis, with the consequence of realizing posterior-oriented comb-like pattern [17]. Notably, primary dendrites of the ddaE neuron extend along the DV direction, which is unlikely affected by the Ten-m signal. Whether its directional growth is also regulated by environmental cues remains uninvestigated.

During embryonic development of *Drosophila*, several morphogens are secreted from the epithelium, providing critical spatial information to govern the morphogenesis of tissues. One morphogen, Wingless (Wg), also known as *Drosophila* Wnt-1, belongs to the Wnt protein family. Wnt proteins have been studied in great detail for their evolutionarily conserved roles in cell fate specification, axon guidance, and synapse formation during the development process of the nervous system [18–20]. Particularly, the Wnt signal has been found to function as an environmental cue in regulating the directional growth of axons, and Frizzled (Fz) and Derailed (Drl)/Ryk receptors have been suggested to mediate attractive and repulsive roles, respectively, in Wnt signaling [21–23]. It has been reported that during dendrite development, Wnt proteins promote dendritic branching and outgrowth in cultured hippocampal neurons of mice as well as in sensory neurons of *C. elegans* [24, 25]. However, whether Wnt signals also function as spatial cues to regulate directional growth of dendrites remains unclear.

In this study*,* we used the *Drosophila* ddaE neuron as an *in vivo* model to study the directional growth of dendrites. We found that epithelia-derived morphogen Wg functions as a repulsive cue to regulate the growth direction of primary dendrites in ddaE neuron.

## Results

### The PD-Angle of the ddaE neuron is decreased when Wg expression is reduced in the adjacent epithelial cells

**Fig. 1:**
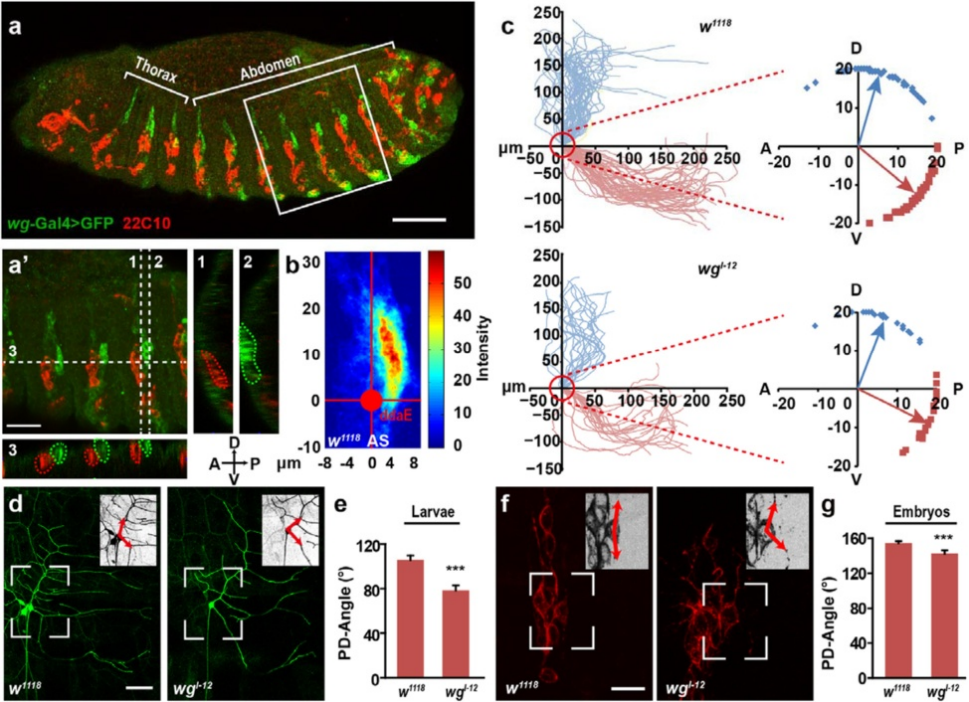
Directional growth of primary dendrites in ddaE neuron is deficient in *wg* ^*l-12*^ mutant. **a** Wg is expressed at the posterior side of the dorsal cluster of da neurons in whole mount fly embryos. Wg expression pattern (green) is monitored by UAS- mCD8-eGFP under the control of *wg*-Gal4. The da neurons (red) are stained by antibody 22C10. White brackets indicate the regions of thorax and abdomen. **a** ***’,*** Enlarged view of AS4-6 (white square in **a**). White broken lines 1–3 indicate the positions of X-Z or Y-Z section images 1–3, respectively. Red broken lines indicate the ddaE neurons and green broken lines indicate the Wg-expression cells. **b** Pseudo-color image shows that Wg-expressing cells are localized mainly in the dorsal-posterior region relative to ddaE neuron in *w* ^*1118*^ AS. **c** The initial 20 μm parts of primary dendrites shift towards AP axis in *wg* ^*l-12*^ mutants. Left panels show the trajectory of primary dendrites of ddaE in the 3^rd^ instar larvae. Red circles indicate the circle around the soma with a radius of 20 μm. The enlarged view of the intersections of the circle and the trajectory of primary dendrites are shown in right panels. Blue dots and red dots indicate the dorsal intersections and ventral ones, respectively. The Blue arrows and red arrows indicate the average direction of dorsal and ventral primary dendrites, respectively. A, P, D and V indicates the direction of anterior, posterior, dorsal and ventral, respectively. (*n* = 24–50). **d**-**e** PD-Angle significantly decreased in *wg* ^*l-12*^ mutants. **d** Representative images of ddaE neurons in wild type *w* ^*1118*^ and *wg* ^*l-12*^ mutant at third instar larvae stage. Gal4^2–21^, UAS-mCD8-eGFP was used to visualize the entire neuron. **e** Quantification statistic of PD-Angle in wild type *w* ^*1118*^ and *wg* ^*l-12*^ mutant. (n = 24–50). **f**-**g** The PD-Angle is decreased in *wg* ^*l-12*^ mutants at embryonic stage 17. (n = 35-57). Scale bars, 50 μm in (**a** and **d**) and 20 μm in (**a** ***’*** and **f**). In all figures, representative images are shown in anterior left and dorsal up; white broken rectangles indicate the position of the inset in the upper-right corner, and red arrows indicate the initial growth direction of the two primary dendrites. *p < 0.05, **p < 0.01, ***p < 0.001, and N.S. indicates no significant changes

To study whether the primary dendrites of the ddaE neuron also develop according to the environmental signals, we examined the localization of epithelia-derived signals relative to the ddaE neuron at the early dendritogenesis stage, i.e. embryonic stage 13–14. Using *wg*-Gal4;UAS-mGFP embryos, we found that in the dorsal region of the abdominal segments 4–6 (AS4-6), Wg was expressed in a small group of epithelial cells adjacent to the dorsal cluster of da neurons (Fig. 1a); however, as shown in the X-Z or Y-Z section images, Wg expression was not detected in those neurons (Fig. 1a’). We set the coordinate system with the position of the ddaE soma as the origin, the AP direction as the X axis, and the DV direction as the Y axis. In this coordinate system, the Wg-expressing cells were mainly localized in the first, i.e. dorsal-posterior quadrant (Fig. 1b). The adjacent localization of Wg expressing cells and the ddaE neuron raises the possibility that Wg functions as a spatial cue to regulate the dendrite patterning of the ddaE neuron.

To determine whether epithelial Wg has an effect on the dendrite growth of ddaE neuron, we used an amorphic mutant *wg*^*l-12*^, the Wg protein of which has a single amino acid change (C104S), resulting in temperature-sensitive deficiency of Wg secretion [26, 27]. To visually display the direction of dendrite extension, we traced the primary dendrites of ddaE neurons and drew a circle around the soma with a radius of 20 μm, and the intersections of primary dendrites and the circle were marked in the AP-DV coordinate system (Fig. 1c). In wild type flies, the intersections of all dorsal primary dendrites were located in the two dorsal quadrants, and those of ventral primary dendrites were mainly located in the ventral-posterior quadrant. In *wg*^*l-12*^ mutant flies, the distribution of dorsal intersections showed little change when compared to wild type flies; however, the ventral intersections greatly shifted towards the AP axis, with a considerable number of those being located in the dorsal-posterior quadrant. To quantify this change in growth direction, we defined the angle between the initial 20 μm of two primary dendrites as the PD-Angle. As expected, the PD-Angle was markedly decreased in the *wg*^*l-12*^ mutant relative to that found in control larvae (Fig. 1d-e). Therefore, these results suggested that epithelial Wg provided a repulsive signal for the directional growth of dendrites.

Dendritogenesis of class I da neurons is initiated during embryonic stage, and the dendrite pattern is stabilized at early larval stages, followed by scaling up during larval development [15, 28]. We thus wondered whether Wg signal affected the directional growth of ddaE neuron from early stages of dendrite development. The main structure of primary dendrites of ddaE neuron is formed by embryonic stage 17. Thus, we examined the dendrite morphology of ddaE at this stage and observed a decrease in the PD-Angle in *wg*^*l-12*^ mutant flies (Fig. 1f-g). These results suggested that the change of PD-Angle in the third instar larval stage was formed at the embryonic stage, during which the Wg signal regulates the directional growth of the primary dendrites, especially the initial parts.

**Fig. 2:**
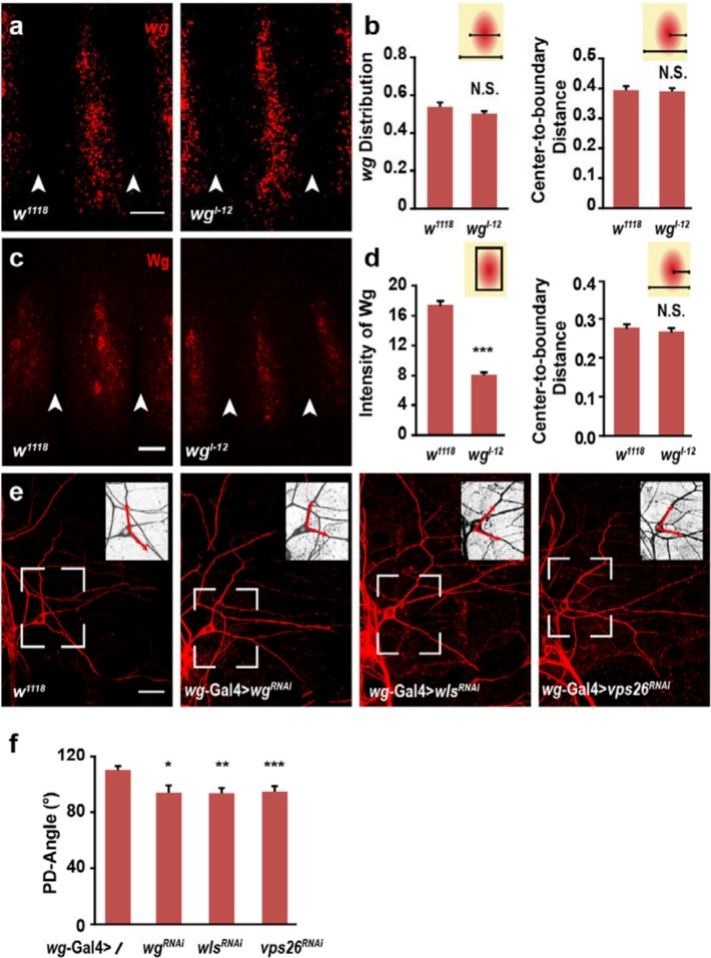
Down regulation of environmental Wg induces the decrease of PD-Angle. **a**-**b** At embryonic stage 14, *wg* RNA is detected by FISH. Normalized to the segment width, both *wg* RNA distribution and the distance between the center of *wg* signal and posterior segmental boundary are comparable to wild type control. White arrow heads in (**a**) indicate the segment boundaries. The insert cartoons in (**b**) represent the expressing region of Wg with red oval, the segment region with yellow rectangle, and the parameters quantified in corresponding panels. (*n* = 15–30). **c** ***-*** **d** Wg expression level is significantly decreased in *wg* ^*l-12*^ embryos, while its center-to-boundary distance is not changed after normalized to the segment boundary. (*n* = 28–32). **e**-**f** Knockdown of *wg*, *wntless* or *vps26* in epithelial cells by *wg*-Gal4 results in the decreased PD-Angle. (*n* = 22–49). Scale bars, 10μm in (**a**) and (**c**) and 25μm in (**c**)

We then examined the expression pattern of Wg in *wg*^*l-12*^ mutant at both mRNA and protein levels by fluorescent *in situ* hybridization (FISH) and antibody immunostaining, respectively. The *wg* mRNA expression was characterized using two parameters, the width of *wg* distribution region (*wg* distribution) and the distance between the center of *wg* distribution and the posterior segmental boundary (Center-to-boundary Distance). To exclude the individual difference in segmental width, we normalized the two parameters to the segmental width in each embryo. Compared to wild type embryos, neither the distribution nor the Center-to-boundary Distance of *wg* mRNA differed in *wg*^*l-12*^ mutant embryos, suggesting that in this mutant, *wg* is expressed in the same group of epithelial cells as those in wild type flies (Fig. 2a-b). To check whether same results apply to Wg protein, we performed Wg antibody immunostaining experiment. As shown in Fig 2c–d, in *wg*^*l-12*^ mutants, however, Wg expression levels were significantly lower, while the Center-to-boundary Distance remained unchanged. This result is consistent with previous reports indicating that the mutation of a signal amino acid in *wg*^*l-12*^ mutant affects the protein stability of Wg but not *wg* mRNA transcription.

To examine whether the dendrite phenotype of ddaE neuron in *wg*^*l-12*^ mutant was affected by reduced Wg expression, we used RNA interference to interrupt Wg signal in wild type flies. Knockdown of *wg* by expressing *wg*^*RNAi*^ in epithelial cells (with *wg*-Gal4) resulted in a decrease in PD-Angle. In contrast, it remained unaffected when *wg*^*RNAi*^ was expressed in ddaE neurons with Gal4^2–21^ (Fig. 2e–f and Additional file 1: Fig. S1). Previous studies indicated that Wntless (Wls) and Vps26 play important roles in Wg secretion process. Down-regulation of Wls and Vps26 results in the accumulation of Wg in the cytoplasm [29, 30]. We found that knockdown of *Wls* and *Vps26* using *wg*-Gal4 resulted in the decreased PD-Angle of ddaE neuron (Fig. 2e–f). These results indicated that down regulating the expression level of Wg or inhibiting the secretion of Wg leads to a more posterior extension of the initial parts of primary dendrites. Taken together, epithelia-derived Wg is posteriorly adjacent to the primary dendrites of ddaE neuron, and reduced Wg signal leads to a significant reduction in the PD-Angle.

### Anterior-shifted Wg also results in dendrite directional growth defect in ddaE neuron

**Fig. 3:**
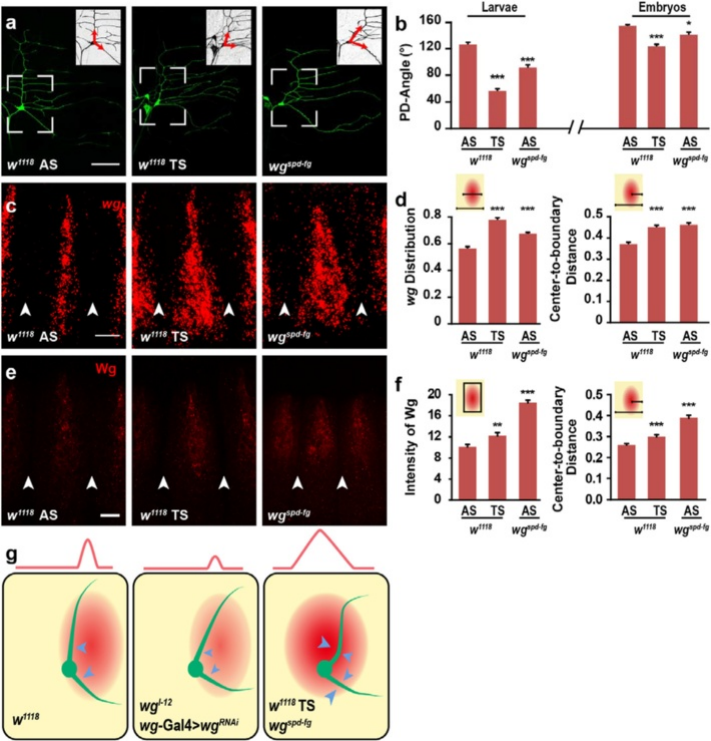
Anterior-shifted Wg expression results in a reduced PD-Angle. **a**-**b** The PD-Angle is significantly decreased in TS and *wg* ^*spd-fg*^ in both late embryos and third instar larvae. (*n* =26- 57). **c**-**d** *wg* expression region is significantly wider in both wild type TS2-3 and *wg* ^*spd-fg*^ AS4-6, when compared to wild type AS4-6. The distance between the center of *wg* signal and posterior segmental boundary is also significantly increased when normalized to segment width. (*n* = 20–29). **e**-**f** Wg expression is markedly increased in TS2-3 of wild-type (*w* ^*1118*^) and in AS4-6 of *wg* ^*spd-fg*^ mutants, and the center of Wg signal is also significantly anteriorly shifted. (*n* = 23-36). **g** Schematic diagram of Wg expression pattern and dendrite orientation. The ddaE neuron is located at the anterior side and adjacent to Wg-expressing cells, and the two primary dendrites are orientated towards the posterior when Wg expression is reduced (as in *wg* ^*l-12*^ and *wg*-Gal4 > *wg* ^*RNAi*^), or when Wg is ectopically expressed in the anterior region (as in *wg* ^*spd-fg*^ and wild-type TS). Scale bar: 50 μm in (**a**) and 10 μm in (**c** and **e**)

When we examined the PD-Angle of ddaE neurons in late embryos or 3^rd^ instar larvae of wild type flies, we noted that in the thoracic segments (TS) of wild type flies, the PD-Angle of ddaE neuron was significantly smaller than that in AS (Fig. 3a–b). To investigate whether this difference is also due to decreased Wg signals as that in the *wg*^*l-12*^ mutant, we next examined the Wg expression patterns in the TS using *wg*-Gal4;UAS-mGFP embryos. We noticed that relative to the Wg-expression pattern in AS, Wg-expressing cells in TS displayed a considerable towards the anterior direction (Additional file 2: Fig. S2a). In agreement with this observation, FISH results indicated that *wg* mRNA in TS was detected in a wider region, and the center of *wg* mRNA signal was shifted towards the anterior direction in stage 13–14 embryos, as indicated by the increased Center-to-boundary Distance when normalized to the segmental width (Fig. 3c–d). Using immunostaining, we found that Wg expression levels were considerably high in TS (Fig. 3e–f). In addition, normalized to segment width, the Center-to-boundary Distance was significantly higher in TS compared to that in AS (Fig. 3f), suggesting that Wg protein was located further towards the anterior end of the TS segment. Together, these results indicated that the PD-Angle is smaller in TS, which might due to the anterior-shifted Wg expression.

To test this possibility, we searched for *wg* mutants with anterior-shifted Wg expression and found a *wg* mutant, namely *wg*^*spd-fg*^. Using immunostaining and FISH, we found that in stage 13–14 embryos, Wg was expressed at a higher level and in a more anterior region in AS of *wg*^*spd-fg*^ mutant compared to wild type embryos. The Wg expression pattern in *wg*^*spd-fg*^ mutant is similar to that in TS of wild type flies (Fig. 3c–f). The *wg*^*spd-fg*^ mutant contains a 1.2kb deletion in the enhancer region of the *wg* gene [31]. Thus, we generate the *wg*^*spd-fg*^-Gal4 transgenic flies to mimic the Wg expression pattern of *wg*^*spd-fg*^ mutant flies. Using GFP as a reporter, we found that in AS4-6 segments of stage 14 embryos, the expression patterns of *wg*^*spd-fg*^-Gal4 shifted anteriorly relative to the soma of the ddaE neuron, when compared with *wg*^*wt*^-Gal4 (Additional file 2: Fig. S2b-c). Together with the results obtained from antibody staining and FISH, we demonstrated that the *wg*^*spd-fg*^ mutant is a *wg* mutant exhibiting anterior-shifted Wg expression, similar to that in TS of wild type flies. In agreement with the result that the PD-Angle of the ddaE neuron is decreased in TS of wild type flies, the PD-Angle of ddaE neuron in *wg*^*spd-fg*^ mutant was also significantly decreased at both embryonic and the 3^rd^ instar larva stage (Fig. 3a-b and Additional file 3: Fig. S3a). Together, these results indicated that the anterior-shifted Wg expression also leads to a decrease in the PD-Angle in both the TS of wild type and the *wg*^*spd-fg*^ mutant.

To further examine whether the abnormal directional growth affects the coverage field of ddaE neuron, we investigated the dendrite coverage area outlined either by the two primary dendrites (PD-Area) or by all branch tips (Total area) in wild type and *wg*^*spd-fg*^ mutant. Both PD-Area and Total area of ddaE neuron were significantly reduced in *wg*^*spd-fg*^ mutant (Additional file 3: Fig. S3b). The coverage range in the DV direction was significantly shorter in *wg*^*spd-fg*^ mutant relative to wild type, but was the same in the AP direction (Additional file 3: Fig. S3c). This suggests that the observed reduction in coverage area was a consequence of the reduction in DV range. Furthermore, the total length of two primary dendrites (PD-total length) was comparable between *wg*^*spd-fg*^ mutant and wild type (Additional file 3: Fig. S3d), suggesting that the decreased coverage area was not caused by general growth defect, but instead by directional growth defect. Together, these findings indicate that the primary dendrites provide the frame of dendritic patterning in ddaE neuron, thus directional growth of primary dendrites significantly contributes to the control of dendrite occupation.

Taken together, our results provide an *in vivo* evidence that Wg signal plays an essential role in regulating the directional growth of primary dendrites in ddaE neuron. Wg is secreted from a small group of epithelial cells posteriorly adjacent to the ddaE neuron. When Wg expression is reduced (as in *wg*^*l-12*^ and *wg*-Gal4 > *wg*^*RNAi*^) or Wg secretion is deficient (*wg*-Gal4 > *wls*^*RNAi*^ and *wg*-Gal4 > *vps26*^*RNAi*^), the extracellular levels of Wg are decreased in the posterior region of the ddaE neuron, resulting in an extension of the two primary dendrites of ddaE neuron to a more posterior position. When the Wg-expressing region is anteriorly shifted (as in *wg*^*spd-fg*^ and TS of wild type), the extracellular Wg levels are increased, especially in the anterior side of the ddaE neuron, and this change also results in the posteriorly-oriented primary dendrites of the ddaE neuron (Fig. 3g). Thus, we hypothesized that the epithelia-derived Wg signal plays a repulsive role in controlling primary dendrite routing of the ddaE neuron. Down-regulation of Wg expression reduces the repulsive power from the posterior side of the ddaE neuron, whereas anteriorly-shifted Wg expression gives the dendrites an anterior to posterior repulsion. Both situations lead to a more posterior directing of the primary dendrites. Furthermore, our results suggest that in neurons with asymmetric dendrite tree like ddaE, the directional growth of the primary dendrites is critical for establishing proper receptive field and thus needs to be precisely regulated.

### Frizzled and Flamingo are required for Wg signal-mediated dendrite routing

**Fig. 4:**
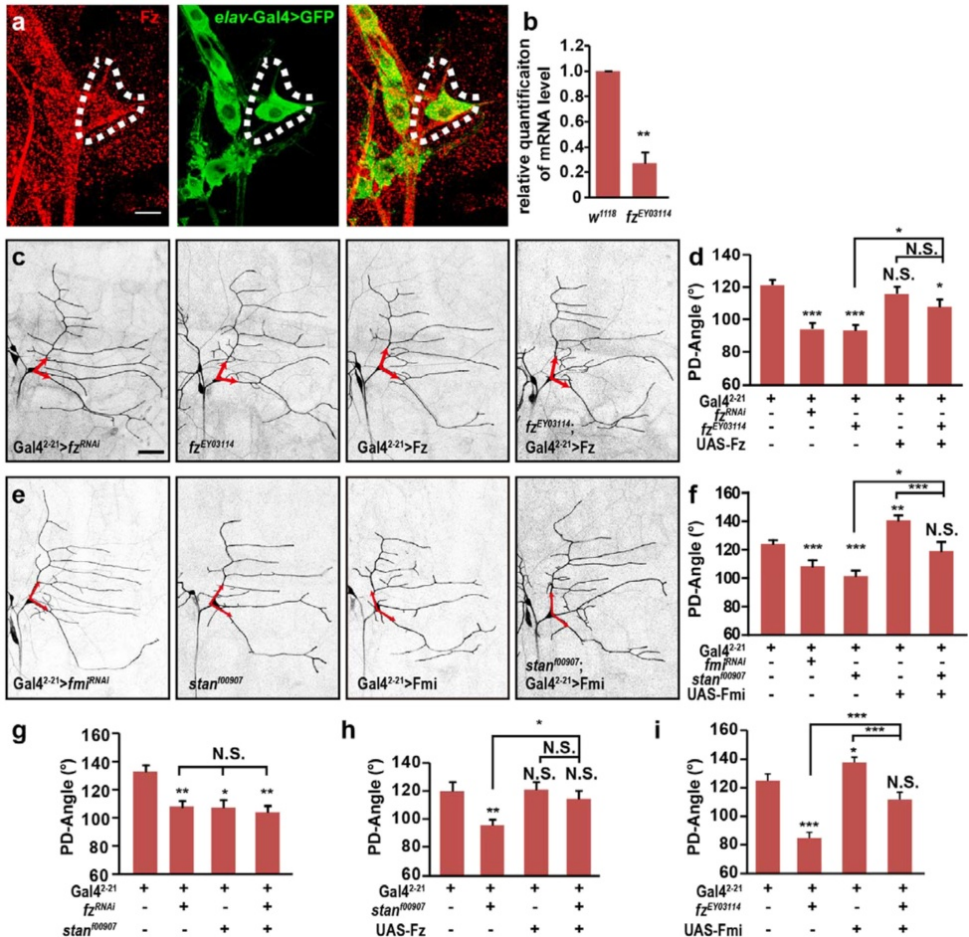
Frizzled and flamingo are required by the ddaE neuron for dendrite directional growth. **a** Fz is expressed in ddaE neuron in the 3^rd^ instar larva. Fz is labeled by Fz antibody (red) and neurons are labeled by elav-Gal4 (green). White dot lines show the soma of ddaE neuron. **b** *fz* expression level is decreased in *fz* ^*EY03114*^ mutant. (*n* = 3). **c**-**d** The PD-Angle is affected by Frizzled. The PD-Angle is significantly decreased in *fz* ^*EY03114*^ hypomorph mutant and neural *fz* ^*RNAi*^ expression larvae. Overexpression of Fz in ddaE neuron does not result in an increase of the PD-Angle. Overexpression of Fz in ddaE neuron in *fz* ^*EY03114*^ mutant background slightly increased the PD-Angle. (*n* = 50–79). **e**-**f** The PD-Angle is affected by Fmi. Knockdown of *fmi* in ddaE leads to decreased PD-Angle, and overexpressing Fmi increases it. Neuronal expressing Fmi rescues the decreased PD-Angle in *stan* ^*f00907*^ mutant. (*n* = 26–50). **g** Knockdown of *fz* in a *stan* ^*f00907*^ mutant background does not further decrease the PD-Angle when compared to *stan* ^*f00907*^ mutant or neural knocking down of *fz*. (*n* = 39–42). **h** Overexpression of Fz in a *stan* ^*f00907*^ mutant background rescues the decreased PD-Angle in *stan* ^*f00907*^ mutant to the wild type degree. (*n* = 32–49). **i** PD-Angle of ddaE neuron with neural overexpression of Fmi in *fz* ^*EY03114*^ mutant background is comparable to the wild type control. (*n* = 26–32). Red arrows indicate the initial parts of primary dendrites. Scale bar: 10 μm in (**a**) and 50 μm in (**c**)

To further investigate how the Wg signal is conveyed into the ddaE neuron, we tested whether the downstream molecules of Wg signal pathway participate in the regulation of the dendrite development. Fz is a classic Wg receptor that plays an essential role in cell polarity and embryonic development [32]. Using immunostaining, we detected the expression of Fz in ddaE neuron in both embryos and the 3^rd^ instar larvae (Fig. 4a and Additional file 4: Fig. S4a). We then employed a *fz* mutant, *fz*^*EY03114*^, to study the function of Fz in regulating dendrite directional growth. In *fz*^*EY03114*^ mutant, a p-element was inserted into the intron of *fz* gene region and the mRNA level of *fz* was significantly decreased as measured by qRT-PCR (Fig. 4b) [33]. Similar to observations in *wg* mutants, a significant decrease of the PD-Angle was found in the *fz*^*EY03114*^ mutant. In addition, when *fz* was knocked down in the neurons by Gal4^2–21^, the PD-Angle was also significantly reduced. Although overexpression of Fz by Gal4^2–21^ showed no increase in PD-Angle in the ddaE neuron, overexpression of Fz in *fz*^*EY03114*^ mutant partially rescued the mutant phenotype (Fig. 4c–d). These results indicated that Fz is required in the ddaE neuron for directional growth during dendrite elaboration.

The Ryk receptor family has emerged as a partner of Wnt in regulating developmental events, like axon guidance [34–36]. We thus examined three members in *Drosophila* Ryk family, Drl, Derailed 2 (Drl-2), and Doughnut (Dnt). The PD-Angle of ddaE neuron was unchanged in any of these mutants or neuronal knockdown larvae (Additional file 5: Fig. S5a–d), indicating that these Ryk receptors are not required for transducing Wg signal in terms of regulating the directional growth of primary dendrites in ddaE neuron.

As an atypical cadherin possessing seven-pass transmembrane receptor features, Fmi is co-localized with Fz and plays an essential role in the PCP pathway [37, 38]. It has been found that Fmi is expressed in the epithelial cells, as well as the da neurons, including their dendrites, to regulate dendrite extension [39, 40]. We adopted two homozygous *fmi* viable mutants, *stan*^*f00907*^and *stan*^*frz3*^, which exhibited reduced Fmi expression in both neurons and epithelial cells (Additional file 6: Fig. S6a) [40, 41]. In these *fmi* mutants, the PD-Angle were significantly decreased, suggesting that similar to Wg and Fz, Fmi is also required for dendrite directional growth (Fig. 4e–f and Additional file 6: Fig. S6b–c). In addition, neuronal knockdown of *fmi* with Gal4^2–21^ also induced a reduction of PD-Angle, while overexpressing Fmi in the neuron significantly increased the PD-Angle. Furthermore, neuronal expression of Fmi in a *stan*^*f00907*^ mutant background fully rescued the decreased PD-Angle in the mutant (Fig. 4e–f), indicating that Fmi plays an essential role in ddaE neuron for regulating dendrite routing.

As both Fz and Fmi were expressed in da neurons at the embryonic stage 14 (Additional file 4: Fig. S4b), we asked whether Fz and Fmi cooperate in dendrite directional growth. Thus we performed genetic interaction experiments between these two molecules, and found that neuronal knockdown of *fz* in the *fmi* mutant *stan*^*f00907*^ background showed comparable PD-Angle either to the *stan*^*f00907*^ mutant itself or to neuronal knockdown of *fz* in wild type background (Fig. 4g). We then performed the rescue experiments in *fz* and *fmi* mutant background, respectively. Neuronal expression of Fz in the *stan*^*f00907*^ mutant was able to rescue the dendrite phenotype to the degree of wild type (Fig. 4h). Similarly, in *fz*^*EY03114*^ mutant background, overexpression of Fmi in ddaE neuron also rescued the PD-Angle to the wild type degree (Fig. 4i). Thus, these results suggested that Fz and Fmi coordinate with each other in regulating directional growth of dendrites.

**Fig. 5:**
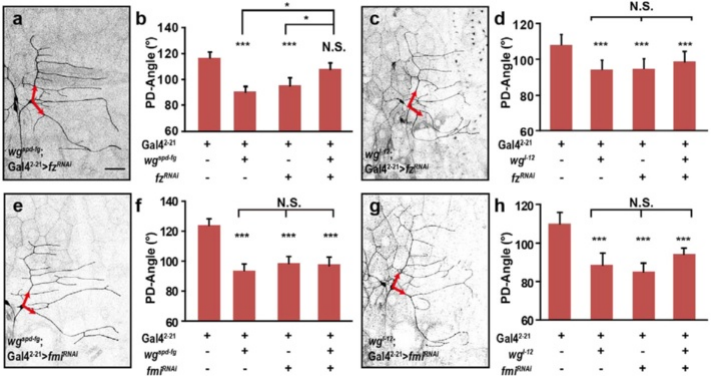
Frizzled and flamingo mediates Wg signal to regulate dendrite directional growth in ddaE neuron. **a**-**d** Knockdown of *fz* in *wg* ^*spd-fg*^ mutant background rescues the PD-Angle to wild type level, while knockdown of *fz* in *wg* ^*l-12*^ mutant background does not further decrease the PD-Angle. (*n* = 25-45). **e**-**h** Knockdown of *fmi* in a *wg* ^*spd-fg*^ or *wg* ^*l-12*^ mutant background does not further decrease the PD-Angle. (*n* = 26-44). Red arrows indicate the initial parts of primary dendrites. Scale bar: 50 μm

To determine whether Fz and Fmi function together in response to Wg signal, we performed genetic interaction experiments between either Wg and Fmi or Wg and Fz. Following knockdown of *fz* in either *wg*^*spd-fg*^ or *wg*^*l-12*^ mutant background, we observed the PD-Angle did not further decrease in both situations. Notably, knockdown of *fz* in a *wg*^*spd-fg*^ background resulted in a significant increase of the PD-Angle when compared to either the mutant or knockdown of *fz* only, while the PD-Angle remained unchanged when knocked down *fz* in *wg*^*l-12*^ mutant background (Fig. 5a–d). In addition, the PD-Angle showed no further reduction following knockdown of *fmi* in either *wg*^*spd-fg*^ or *wg*^*l-12*^ background (Fig. 5e–h). Together, these results suggested that neuronal Fz and Fmi cooperated in responding to environmental Wg and mediating the downstream events of Wg signal.

### Dishevelled and Rac1, but not main members of the β-catenin pathway, are required for conveying Wg signaling

**Fig. 6:**
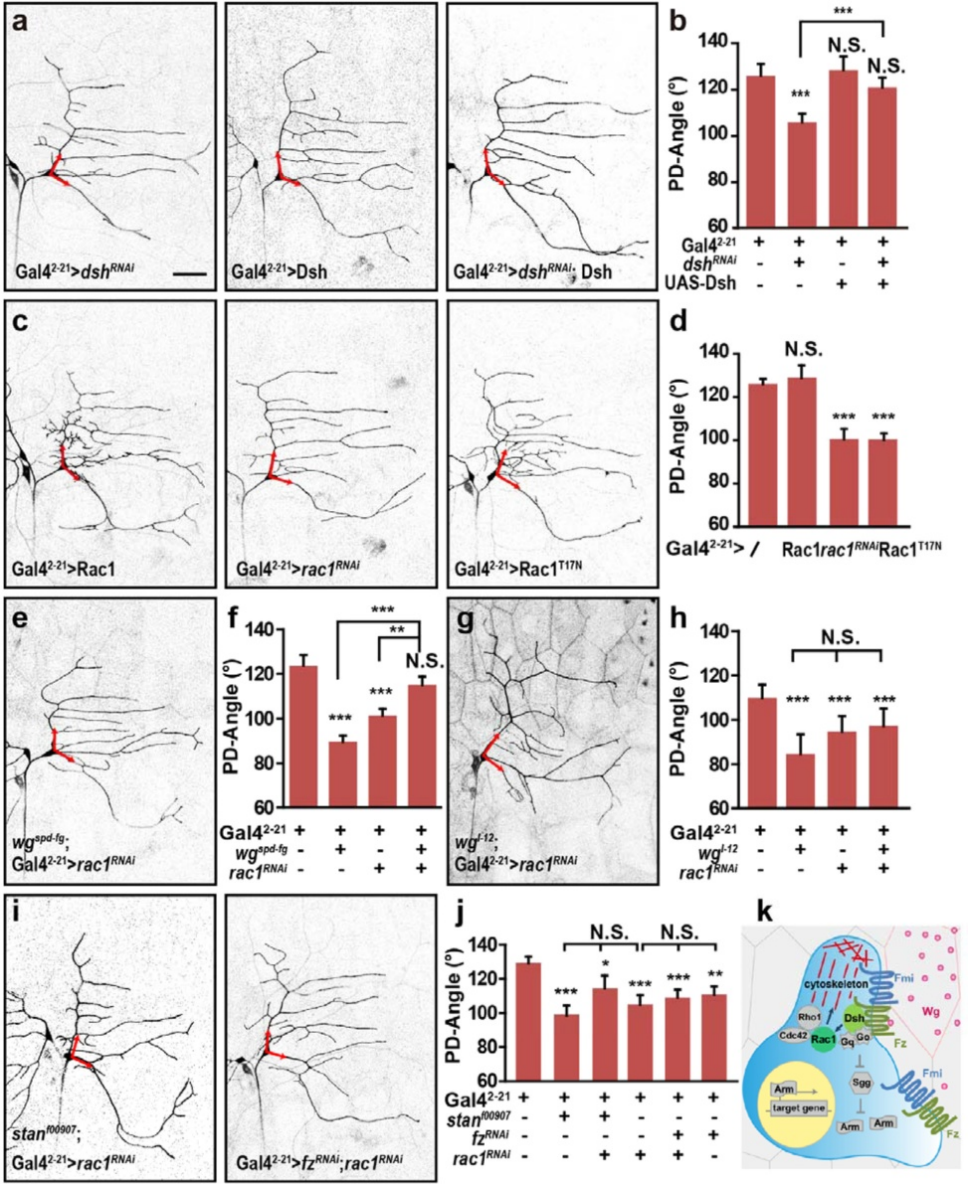
Dishevelled and Racl are involved in regulating dendrite orientation. **a**-**b** Knockdown of *dsh* results in a significant reduction in the PD-Angle, which is rescued by expressing Dsh in the neuron. (*n* = 24–46). **c**-**d** The PD-Angle is decreased when Rac1 is suppressed by expressing either *rac1* ^*RNAi*^ or Rac1^T17N^ (a dominant negative form), whereas overexpressing Rac1 shows no effect on the PD-Angle. (*n* = 38–53). **e**-**f** Knockdown of *rac1* partially rescues the reduced PD-Angle in *wg* ^*spd-fg*^ mutant background. (*n* = 59–77). **g**-**h** Knockdown *rac1* does not further decrease the PD-Angle in *wg* ^*l-12*^ mutant background. (*n* = 24–36). **i**-**j** Knockdown of *rac1* does not lead to further change of the PD-Angle in either *fmi* mutant *stan* ^*f00907*^ or *fz* Knockdown of background. (*n* = 37–76). **k** Schematic diagram of molecules in Wg signal pathway that regulates dendrite orientation. Fz, Fmi, Dsh, and Rac1 are essential for mediating Wg signal to regulate directional growth of dendrites, while G proteins, β-catenin pathway, and Cdc42/Rho1 are dispensable for the dendrite directional growth of ddaE neuron. Red arrows indicate the initial parts of primary dendrites. Scale bar: 50 μm

Dsh is a key molecule for both canonical and non-canonical Wnt signaling, where it functions downstream of Fz and Fmi [42, 43]. Knockdown of *dsh* in ddaE neuron led to a significant reduction in the PD-Angle, which was rescued by neuronal expressing Dsh (Fig. 6a–b). Thus, downstream of Fz and Fmi, Dsh is also required for the regulation of dendrite directional growth in ddaE neuron.

The canonical Wnt signal pathway is mediated by GSK3β and β-catenin [44], the *Drosophila* homologous of which are *shaggy* (*sgg*) and *armadillo* (*arm*), respectively. G protein Go mediates both Wg and PCP pathways transduced by Fz [45–47]. In addition, another G protein Gq has been suggested to couple with Fz in *Drosophila* [46] and also has been shown to function as a downstream molecule of Fmi intercellular signal in repressing dendrite growth [40]. However, we found that neither knockdown nor expressing a dominate-negative form of these proteins in ddaE neurons affected the PD-Angle, except for the forced expression of the transcription factor Arm, which could interfere with the expression of amount of Arm targeting genes (Additional file 7: Figure S7a–b). These results suggest that the canonical Wnt pathway is not required for mediating Wg signal in dendrite routing.

Small Rho GTPases have been shown to play critical roles in cytoskeleton assembly and neuronal morphogenesis [48, 49]. We therefore examined whether Rac1, Cdc42, and Rho1 play a role in dendrite directional growth employing both gain-of and loss-of-function. The results showed that the PD-Angle was reduced when Rac1 was suppressed by neuronal expressing either *rac1*^*RNAi*^ or Rac1^T17N^ (a dominant negative form) (Fig. 6c–d). In contract, neither activating nor suppressing the activity of Cdc42 or/and Rho1 affect the PD-Angle (Additional file 7: Fig. S7c). Together, these results suggested that the small Rho GTPase Rac1 participates in the regulation of dendrite directional growth, which might function as a downstream factor in transducing the Wg signal to the cytoskeleton.

To examine whether Rac1 functions downstream of Wg signaling, we knocked down *rac1* in either a *wg*^*spd-fg*^ or *wg*^*l-12*^ mutant background. Knockdown of *rac1* in *wg*^*spd-fg*^ background, led to a statistically increase of the PD-Angle when compared to either the mutant or knockdown of *rac1* only, while the PD-Angle remained unchanged following knockdown of *rac1* in a *wg*^*l-12*^ background (Fig. 6e-h). These results suggested that in the *wg*^*spd-fg*^ mutant, at the position of ddaE neuron, the extracellular Wg distribution is changed, and down regulating *rac1* may weaken the repulsive effect of Wg signal, resulting in a recovery of PD-Angle. Similar result was abstained from the interaction experiment of *wg*^*spd-fg*^ and *fz*. Furthermore, the PD-Angle remained unchanged following knockdown of *rac1* in either *fmi* mutant *stan*^*f00907*^ or *fz* knockdown of background (Fig. 6i–j). Thus, these results suggest that Rac1 functions downstream of Fz and Fmi, thereby linking Wg signaling to the cytoskeleton, and this regulation is essential for proper directional growth of dendrites.

## Discussion

The shape of dendrites is a major factor in determining both neuron morphology and the receptive field of dendrites, and therefore plays an important role in proper physiological functioning of neurons. Here, we report that epithelial Wg signal functions as a repulsive cue to control the growth direction of primary dendrites. We tested several Wg receptors and downstream molecules, and found that Fz, Fmi, Dsh, and Rac1 are essential for dendritic directional growth in response to Wg signals. The requirement of Rac1 suggests that the local regulation of cytoskeleton is required for dendrite routing. In contrast, the redundancy of β-catenin Wnt pathway suggests that the transcriptional control is not responsible for directional growth of dendrites (Fig. 6k). Our findings provide *in vivo* evidence that dendrites utilize Wnt signal as a spatial cue to generate a desired dendrite pattern.

### Wingless plays a repulsive role in the directional growth of primary dendrites

The function of Wnt signaling in neuronal morphogenesis has been widely studied in the nervous system. Evolutionary conserved roles of Wnts are reported as both attractive and repulsive roles for regulating the direction of axon growth and guidance [21, 36, 50]. Wnt signal has been reported to promote dendrite branching and growth in cultured hippocampal neurons and *C. elegans*, respectively [24, 25]. Nevertheless, the regulatory mechanisms of Wnt signaling in directional growth of dendrites have not been elucidated.

Here, we demonstrate that epithelial Wg provides repulsive cues for the primary dendrites of ddaE neuron. We employed two homozygous *wg* mutants, *wg*^*l-12*^ and *wg*^*spd-fg*^. Reduction in PD-Angle was observed in both of these two mutants. However, the W*g* expression patterns in these two mutants are different. Wg expression was decreased in the posterior part in *wg*^*l-12*^ mutant, and increased in the anterior part in *wg*^*spd-fg*^. In both situations, the change of the primary dendrites in direction relative to the body axes indicates that Wg signal functions as a repellent in regulating directional growth of dendrites, but not an inhibitor of general growth. Together, these results suggest that in addition to the function of Wnt signal in regulating axon guidance and dendrite outgrowth, this signal also serves as an important spatial cue for directional growth of dendrites.

### The directional growth of primary but not secondary dendrites of ddaE neuron is regulated by epithelial Wg signal

The ddaE neuron features dendrites of comb-like pattern, with both primary and high-order dendrites extending in fixed directions. The cell-autonomous functions of various transcriptional factors in regulating the pattern of ddaE neuron have been studied in great details [51–53]. Environmental cues also participate in the dendrite patterning process. The neuron-glia interaction mediated by Neuroglian is critical for the formation of secondary order dendrites of ddaE neuron [54]. A recent study showed that during dendrite directional growth, Ten-m is expressed in both neurons and epithelial cells, and the homodimer interaction guides the posterior extension of secondary dendrites according to the Ten-m gradient along the AP direction [17]. Our results showed that epithelia-derived Wg plays a repulsive role in regulating the routing of primary dendrites. Notably, although the primary dendrites grow outside Wg-expressing area, secondary dendrites grow straight through that region.

To understand the different responses between primary and secondary dendrites, we analyzed the detailed time window of Wg expression. The primary dendrites of ddaE neuron start sprouting at 13 h after egg laying (AEL), when Wg is still expressed in adjacent epithelial cells. In contrast, the secondary dendrites of ddaE neuron emerge at the end of embryogenesis, later than 15 h AEL, following the decay of Wg expression to levels below the detection limit. Thus, the time window of Wg expression allows its specific regulation in the directional growth of primary dendrites but not the secondary dendrites. Taken together with the findings that Ten-m signal specifically regulates the directional growth of high order dendrites, our findings show that epithelial signals line the dendrites at both DV and AP directions, and this regulation is precisely programmed during development.

### Frizzled and Flamingo cooperate in mediating the Wg signal in the regulation of dendrite directional growth

The 7-transmembrane protein Fz and Fmi are core components of planar cell polarity (PCP) pathway. Previous studies indicate that Fz physically interacts with Fmi to mediate intercellular polarity signaling [37, 38]. Besides the function in PCP pathway, Fmi and Fz have also been found to play important roles in neural development. In studies on invertebrate, Fmi was reported to regulate axon and dendrite development in a Fz-independent manner [39, 40, 55–58]. In mammalian system, the interaction of Fz and Fmi was proposed to modulate the axon guidance in the commissural neurons and intrinsic enteric neurons [59, 60]. Thus, whether these two molecules also function together in the nervous system remains unclear.

In this study, we demonstrate both Fz and Fmi functions in Wg signaling to regulate dendrite directional growth. Our results showed that simultaneous down regulation of both *fz* and *fmi* did not further decrease PD-Angle. Moreover, overexpression of Fz in *stan*^*f00907*^ mutant background fully rescued the decreased PD-Angle to the degree of wild type. In the reverse case, neural overexpression of Fmi in *fz*^*EY03114*^ mutant also rescued the mutant phenotype with no statistic difference to the wild type; however, the average PD-Angle in the rescue group was slightly smaller than wild type. Thus, we suspect that Fz, as a known Wg receptor, plays the key role in directing dendrite growth, while Fmi cooperates with Fz in this process. The rescue effect of Fmi overexpression may be based on the residue expression of Fz in *fz*^*EY03114*^ mutant (approximately 20% of wild type as shown in Fig. 4b). Previous studies have showed that in da neurons, Fmi regulates dendrite growth independent of Fz [39, 40]. Thus, our findings suggest that Fz-dependent function of Fmi specifically contributes to the control of directional growth but not general growth of dendrites.

### Directional growth of primary dendrites is regulated by non-canonical Wnt pathway through Dsh and Rac1

As a key component of Wnt signaling, Dsh is required for mediating both the canonical β-catenin pathway and the non-canonical pathway. In this study, Dsh was also found to participate in the Wg signal-mediated directional growth of primary dendrites. Furthermore, we found that downstream of Fz-Fmi-Dsh, the canonical Wnt/β-catenin pathway is dispensable for dendrite directional growth; instead, small GTPases Rac1 plays a critical role. It has been reported that Rac1 functions as a downstream factor in non-canonical Wnt pathway and regulates the dynamic of actin cytoskeleton in axon guidance and dendrite branching [25, 61, 62]. In particular, in response to Wnt signal, Rac1 has been found to directly bind to Dsh and regulate dendrite branching in cultured hippocampal neurons [25]. Remarkably, another two small GTPase, Cdc42 and Rho1, are not activated by Wnt7a in this response. In agreement, our *in vivo* results show that Rac1 is the effecter downstream of Wg signaling in dendrite routing, while Cdc42 and Rho1 are dispensable. Therefore, our results indicate that in the ddaE neuron, downstream of Fz-Fmi-Dsh, Rac1 conducts the Wg signaling to cytoskeleton, thereby regulating the directional growth of dendrites.

## Conclusions

In conclusion, we established PD-Angle as a new parameter to analyze dendrite directional growth, and found that epithelial Wg serves as a repulsive cue that functions through Fz, Fmi, Dsh, and Rac1 to regulate the directional growth of primary dendrites. Thus, these findings gain insights into the non-cell-autonomous regulation by environmental cues of dendrite routing in neural development.

## Methods

### Fly stocks

All flies were maintained at 25°C, except that flies containing *wg*^*l-12*^ mutant were raised at 17°C. Fly strains of Gal4^2–21^, *wg*-Gal4, and *elav*-Gal4 were kindly provided by Dr. Fen-Biao Gao (University of Massachusetts Medical School). UAS-Fmi was a gift from Dr. Tadashi Uemura (Kyoto University). UAS-Go^GTP^ was from Dr. Andrew Tomlinson (Columbia University). UAS-Arm was generously provided by Dr. Haiyun Song (Shanghai Institutes for Biological Sciences, CAS). UAS-*wntless*-RNAi and UAS-*vps26*-RNAi were generously provided by Dr. Peng Zhang and Dr. Zengqiang Yuan (Institutes of Biophysics, CAS). The following fly strains were from the Bloomington stock center: *fmi* mutants *stan*^*f00907*^ and *stan*^*frz3*^, *wg* mutants *wg*^*spd-fg*^ and *wg*^*l-12*^, *fz* mutant *fz*^*EY03114*^, UAS-mCD8-eGFP, UAS-Dsh^myc^, UAS-Sgg^S9E^, UAS-Sgg^B^, UAS-Rac1^T17N^, UAS-Rac1, UAS-Rho1, UAS-Rho1^G14V^, UAS-Rho1^T19N^, UAS-Cdc42^T17N^, UAS-Cdc42, UAS-Gα49B^R^, UAS-Gα49B^dsRNA.1f1^, UAS-Gq^*RNAi*^, UAS-*fmi*^*RNAi*^, UAS-*rac1*^*RNAi*^, and UAS-*cdc42*^*RNAi*^. The following RNAi fly lines were from Vienna *Drosophila* RNAi Center (VDRC): UAS-*wg*^*RNAi*^, UAS-*dsh*^*RNAi*^, UAS-*sgg*^*RNAi*^, and UAS-Go^*RNAi*^. UAS-*arm*^*RNAi-1*^ and UAS-*fz*^*RNAi*^ were gifted from Dr. Jian-Quan Ni (Tsinghua Fly Center, School of Medicine, Tsinghua University). UAS-*arm*^*RNAi-2*^ was obtained from Fly Stocks of National Institute of Genetics (NIG-FLY).

The *wg*^*spd-fg*^-Gal4 construct was generated by subcloning a 9kb DNA fragment upstream of the *wg* coding region from the genomic DNA of the *wg*^*spd-fg*^ mutant into a pPTGal vector [63]. The primers for the promoter amplification were 5’-AAAAGGCCTGTACTTTGAATCTTTCACCTGCG-3’, 5’-CGGGGTACCTATTGCTGATCGGGTTTATCTGTT-3’. We also used these primers to amplify the promoter of the *w*^*1118*^ flies and generated the *wg*^*wt*^-Gal4 as a control.

### Immunohistochemistry

Embryos and body walls of third instar larvae were immunostained according to the standard protocol [64] and was described in details in a previous research of our lab [40]. Samples were incubated in primary and then secondary antibodies at 4°C over night. Rabbit-anti-Fz antibody (1:300) was kindly provided by Dr. David Strutt (University of Sheffield). Mouse monoclonal antibodies were purchased from Developmental Studies Hybridoma Bank (DSHB, University of Iowa): Wg (4D4, 1:250), Fmi (74-C, 1:100), 22C10 (1:500). Secondary antibodies were Alexa Fluor® 555 Goat anti-mouse IgG (Invitrogen A21422, 1:1000), Alexa Fluor® 488 Goat anti-rabbit IgG (Invitrogen A11008, 1:200) and Alexa Fluor® 555 Goat anti-rabbit IgG (Invitrogen A21428, 1:200).

### Fluorescent *In Situ* Hybridization (FISH)

Embryos of stage 13–14 were collected and fixed according to standard procedures [65]. The FISH was performed by using RNA scope based signal amplification (Advanced Cell Diagnostics). The *wg* probe was designed to target the 479–1474 nt of *wg* mRNA and labeled with C2 color channel. After rehydration, embryos were post-fixed for 25 min by 5% formaldehyde in PBT and the protease digested using Protease III for 5min, following a second post-fixation. The probe hybridization was performed at 40°C O/N, and the RNA signal was amplified by Amp1-4 at 40°C. After each hybridization step, embryos were washed by 0.02% SSCT. Amp 4 Alt A-FL was used for the fluorescent labeling.

### Imaging and analysis

VECTASHIELD® mounting medium (Vector Laboratories) was used to mount samples after immunostaining. Third instar larvae were rinsed in PBS and then immobilized using a cover slip for *in vivo* imaging. Confocal images were obtained with a Leica SPE or a SP5 II MP microscope or a Nikon A1 confocol. The dendrites morphology was analyzed using NIS-Element D 3.0 (Nikon) and a self-developed program, and the fluorescent intensity was measured by Image J (National Institutes of Health, USA).

The parameters for estimating the dendrite morphology of ddaE neuron were defined as: 1) PD-Angle, the angle between two primary dendrites, which is determined by initial 20 μm primary dendrites; 2) the initial parts of primary dendrites, the trajectory of primary dendrites of ddaE neuron was traced by ImageJ and plotted by R project (R Development Core Team), the 20 μm points were dotted by ImageJ and plotted in Excel (Microsoft), and the coordinate of average direction of dorsal or ventral primary was the average of the coordinates of all dorsal or ventral intersections; 3) PD-Area, the dendrite area outlined by the primary dendrites; 4) Total area, the dendrite area outlined by all dendritic tips; 5) The dendritic coverage range at AP direction and at DV direction; 6) PD-Total length, the total length of the two primary dendrites. Neurons that were not able to establish the entire primary dendrites were not included in the data analysis.

To examine the expression of Wg signal, we conducted the following experiments: 1) Wg-expressing cells were visualized using UAS-mCD8-eGFP driven by *wg*-Gal4 or *wg*^*spd-fg*^-Gal4 or *wg*^*wt*^-Gal4 signal, and neurons were stained by 22C10 antibody. In Fig. 1b and Additional file 3: Fig. S3a, 25 images of AS4-6 and 20 images of TS2-3 were obtained from 10 *wg*-Gal4 > GFP embryos. In Additional file 3: Fig. S3c, 18 images of AS4-6 were obtained from 10 *wg*^*spd-fg*^-Gal4 > GFP or *wg*^*wt*^-Gal4 > GFP embryos. All images were rotated to anterior-left and dorsal-up and were aligned by having ddaE neuron on the origin of the coordinate. The gray value of GFP signal was read, averaged and calculated to generate pseudo-color figures by Matlab (MathWorks, Natick, MA). Showing in Fig. 1b and Additional file 3: Fig. S3, different colors represented the corresponding brightness as the bar indicated. 2) Intensity of Wg, after standard Wg antibody staining and confocal imaging procedure, the fluorescent intensity in a 10 × 30 μm^2^ region covering all Wg-expressing cells in the dorsal side of a semi-segment is measured and averaged by Image J; 3) Center-to-boundary Distance, the distance between the center of Wg or *wg* RNA signal and the posterior segmental boundary, which is normalized to segment width; 4) *wg* distribution, the range of *wg* mRNA distribution at A-P axis normalized to segment width. During the measurement of the parameter Center-to-boundary and *wg* distribution, the genotypes were renumbered and blinded to the operator.

Data in this paper met to normal distribution. Statistical analysis was performed using two tails Student’s t-test and one-way analysis of variance (ANOVA) with Tukey’s test as a post hoc comparison. Quantification data are shown in mean ± s.e.m; *P < 0.05, **P < 0.01, ***P < 0.001, and N.S. represents no significant.

### Quantitative real time PCR

Total RNA was extracted from approximate 50 ul embryos at embryonic stage 14 with TRIzol (Invitrogen). 1μg of RNA samples were treated with RQ1 DNase (Promega) and reverse transcribed using PrimeScript RT Master Mix (TaKaRa). Relative quantification PCR was carried out using a SYBR Premix Ex TaqTM II kit (Takara) and an ABI PRISM 7300 real-time PCR Detection system (Applied Biosystems). Relative mRNA levels were calculated using the comparative CT method. Rp49 was used as an internal control, and gene expression levels were normalized to treatment control or genetic control. Three separate samples were collected from each condition, and measurements were conducted in triplicates. The primers of *fz* for q-PCR were *fz*-qPCR-exon1-F: TGCAACTGAAAACGCCTCT; *fz*-qPCR-exon2-R: AAACGGCCAA GAAGACAATG. The primers of *rp49* were *rp49*-RT-F: AGGGTATCGACAACAGAGTG; *rp49*-RT-R: CACCAGGAACTTCTTGAATC

### Ethics approval and consent to participate

The research does not involve in any human subjects. All the animal experiments are in accordance with ethical principles of Basel Declaration and ethical guidelines of the International Council for Laboratory Animal Science.

### Statement of consent for publication

This manuscript contains no individual person’s data. The statement of consent for publication is not applicable in this article.

### Availability of data and materials

The datasets supporting the conclusions of this article are included within the article and its additional files.

## Additional file

Additional file 1: Fig S1. Knockdown of *wg* by Gal4^2–21^ in ddaE neuron has no effect on PD-Angle. **a**, Representative images of ddaE neurons in control and UAS-*wg* ^*RNAi*^/+;Gal4^2–21^/+ larvae. **b**, Quantification statistic of PD-Angle in (**a**). White arrows indicate the initial parts of primary dendrites. n ≥ 30 in each group. Scale bar, 50 μm. (TIF 340 kb) Additional file 2: Fig S2. Wg expression region is anteriorly shifted in TS of *wg*-Gal4 and *wg* ^*spd-fg*^-Gal4. **a**, Wg-expressing cells are localized anterior-shifted in TS of wild type flies when comparing to AS. Red dots indicate the ddaE neurons. **b**-**c** ***,*** Wg-expressing cells are localized is anterior-shifted in AS4-6 of GFP labeled *wg* ^*spd-fg*^-Gal4 embryos. **b**, The represent images of the expression pattern of *wg* ^*wt*^-Gal4 and *wg* ^*spd-fg*^-Gal4. The Gal4 expression regions were shown by GFP (green). The da neurons were labeled by 22C10 antibody (red). Red broken circles indicate the ddaE neurons. **c**, the pseudo-color figures shows the average Gal4 expression region of *wg* ^*wt*^-Gal4 and *wg* ^*spd-fg*^-Gal4 relative to ddaE neuron. Red dots indicate the ddaE neurons. Scale bar: 5 μm. (TIF 954 kb) Additional file 3: Fig S3. Dendrite coverage area and coverage range at DV direction of ddaE neuron are statistically decreased in *wg* ^*spd-fg*^ mutants. **a**, The initial 20 μm parts of primary dendrites shift towards AP axis in *wg* ^*spd-fg*^ mutants. The data are obtained and presented in the same way as in Fig. 1c. **b**-**d** ***,*** The PD-Area or Total area (**b**), and the dendritic coverage range at DV direction (**c**) are significantly decreased in wg^spd-fg^ mutants, while the PD-Total length (**d**) and the dendritic coverage range at AP direction (**c**) are unchanged. The insert cartoons show dendrites in green lines and the parameter quantified in corresponding panels. (*n* =42-43). (TIF 399 kb) Additional file 4: Fig S4. Fz was expressed in ddaE and the expression level decreased in *fz* ^*EY03114*^ mutant. **a**, Fz expression is detected in ddaE neuron in wild type S14 embryos. Fz protein is shown by anti-Fz antibody (green) and neurons are shown by antibody 22C10 (red). **b**, Fz is co-expressed with Fmi in dorsal cluster da neurons. Fz protein is shown by anti-Fz antibody (green) and neurons are shown by anti-Fmi antibody (red). Scale bar: 5 μm. (TIF 1.11 mb) Additional file 5: Fig S5. The PD-angle is not affected in RYK-family receptor drl, drl-2, or dnt mutant or knock-down flies. **a**-**b**, No significant change of PD-Angle is observed in *drl*, *drl-2*, or *dnt* mutants. **c**-**d**, Neuronal overexpression or knock-down of either *drl* or *drl-2* has no effect on the PD-Angle. White arrows indicate the initial parts of primary dendrites. *n* ≥ 30 in each group. Scale bar, 50 μm. (TIF 1.44 mb) Additional file 6: Fig S6. Flamingo is required in ddaE neuron for dendrite directional growth. **a**, In homozygous available *stan* ^*frz3*^ mutant, Fmi expression is decreased in epithelia and da neurons. **b**-**c** ***,*** The PD-Angle is significantly decreased in *stan* ^*frz3*^ mutant. White arrows indicate the initial parts of primary dendrites. *n* ≥ 30 in each group. Scale bar, 5 μm in (**a**) and 50 μm in (**b**). (TIF 1.05 mb) Additional file 7: Fig S7. Canonical-Wnt pathway and small GTPase Cdc42, Rho1 were unaffected in dendrite directional growth. **a**, Neither knockdown nor overexpression of Go or Gq in ddaE neurons has an effect on the PD-Angle. **b**, Neither knockdown nor overexpression of Sgg or Arm in ddaE neurons has an effect on the PD-Angle, except that overexpressing Arm results in a significant decrease. **c**, No significant change of the PD-Angle is shown when manipulating Cdc42 or/and Rho1 in ddaE neuron. CA, constitutively active form; OE, overexpression; DN, dominant negative form. *n* ≥ 30 in each group. (TIF 349 kb)

## Abbreviations

AEL: after egg laying
AP: anterior-posterior
Arm: Armadillo
AS: abdominal segments
BDNF: Brain-derived neurotrophic factor
Center-to-boundary Distance: the distance between the center of *wg* distribution and the posterior segmental boundary
da: dendritic arborization
Dnt: Doughnut
Drl: Derailed
Drl-2: Derailed 2
Dsh: Dishevelled
DV: dorsal-ventral
ECM: extracellular matrix
FISH: fluorescent *in situ* hybridization
Fmi: Flamingo
Fz: Frizzled
PCP: planar cell polarity
PD-Angle: the angle between the initial 20 μm of two primary dendrites
PD-Area: the dendrite coverage area outlined by the two primary dendrites
PD-Total length: the total length of the two primary dendrites
Sema3A: Semaphorin3A
Sgg: Shaggy
Total area: the dendrite coverage area outlined by all branch tips
TS: thoracic segments
Wg: Wingless
*wg* distribution: the width of *wg* distribution region
Wls: Wntless
