# Supplementary figures and images for: Epithelia-derived wingless regulates dendrite directional growth of drosophila ddaE neuron through the Fz-Fmi-Dsh-Rac1 pathway
Source: Mol Brain. 2016 Apr 29;9:46. doi: 10.1186/s13041-016-0228-0 (PMC4850637; doi:10.1186/s13041-016-0228-0)

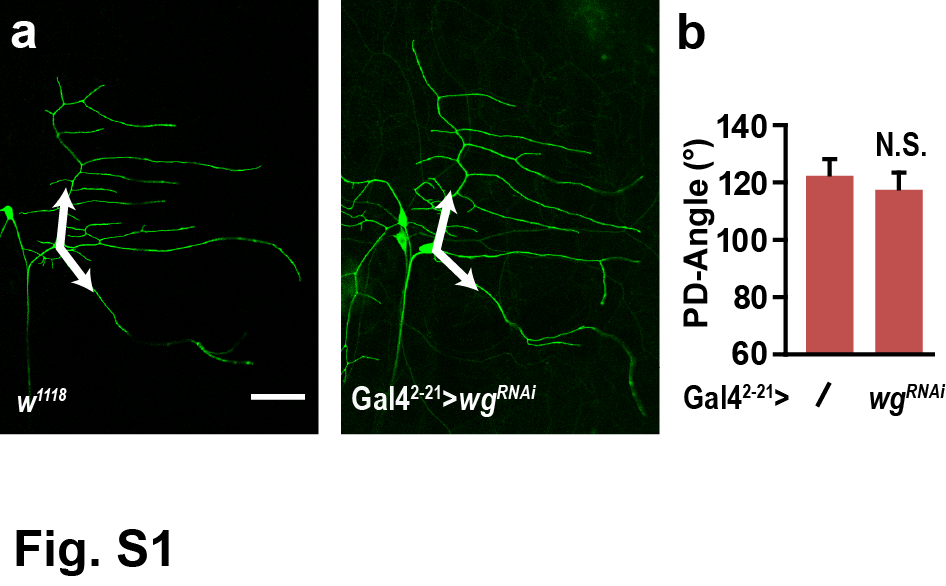

Supplement: Additional file 1: Fig S1. — Knockdown of wg by Gal42–21 in ddaE neuron has no effect on PD-Angle. a, Representative images of ddaE neurons in control and UAS-wg RNAi/+;Gal42–21/+ larvae. b, Quantification statistic of PD-Angle in (a). White arrows indicate the initial parts of primary dendrites. n ≥ 30 in each group. Scale bar, 50 μm. (TIF 340 kb) [file 13041_2016_228_MOESM1_ESM.tif]

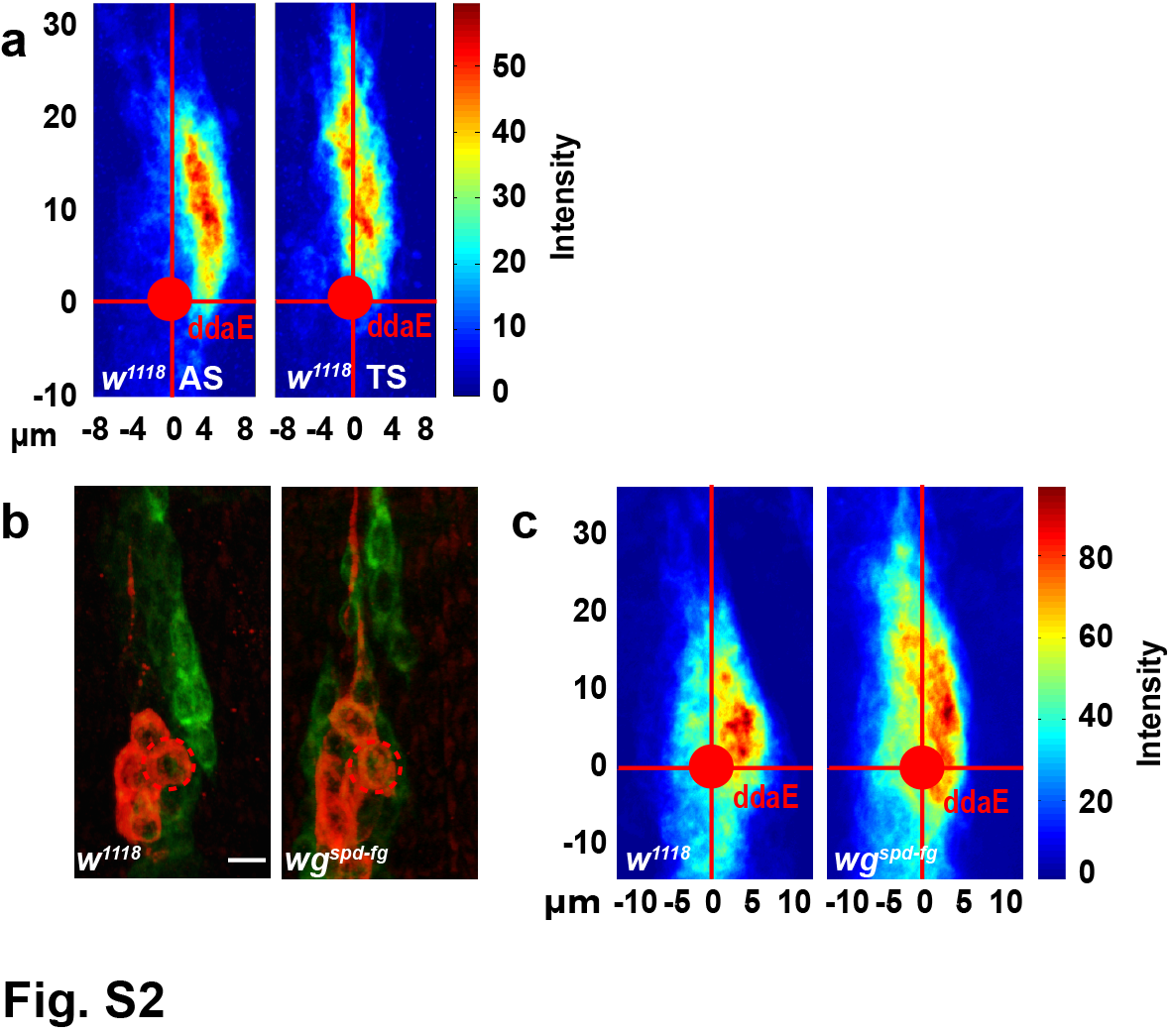

Supplement: Additional file 2: Fig S2. — Wg expression region is anteriorly shifted in TS of wg-Gal4 and wg spd-fg-Gal4. a, Wg-expressing cells are localized anterior-shifted in TS of wild type flies when comparing to AS. Red dots indicate the ddaE neurons. b-c , Wg-expressing cells are localized is anterior-shifted in AS4-6 of GFP labeled wg spd-fg-Gal4 embryos. b, The represent images of the expression pattern of wg wt-Gal4 and wg spd-fg-Gal4. The Gal4 expression regions were shown by GFP (green). The da neurons were labeled by 22C10 antibody (red). Red broken circles indicate the ddaE neurons. c, the pseudo-color figures shows the average Gal4 expression region of wg wt-Gal4 and wg spd-fg-Gal4 relative to ddaE neuron. Red dots indicate the ddaE neurons. Scale bar: 5 μm. (TIF 954 kb) [file 13041_2016_228_MOESM2_ESM.tif]

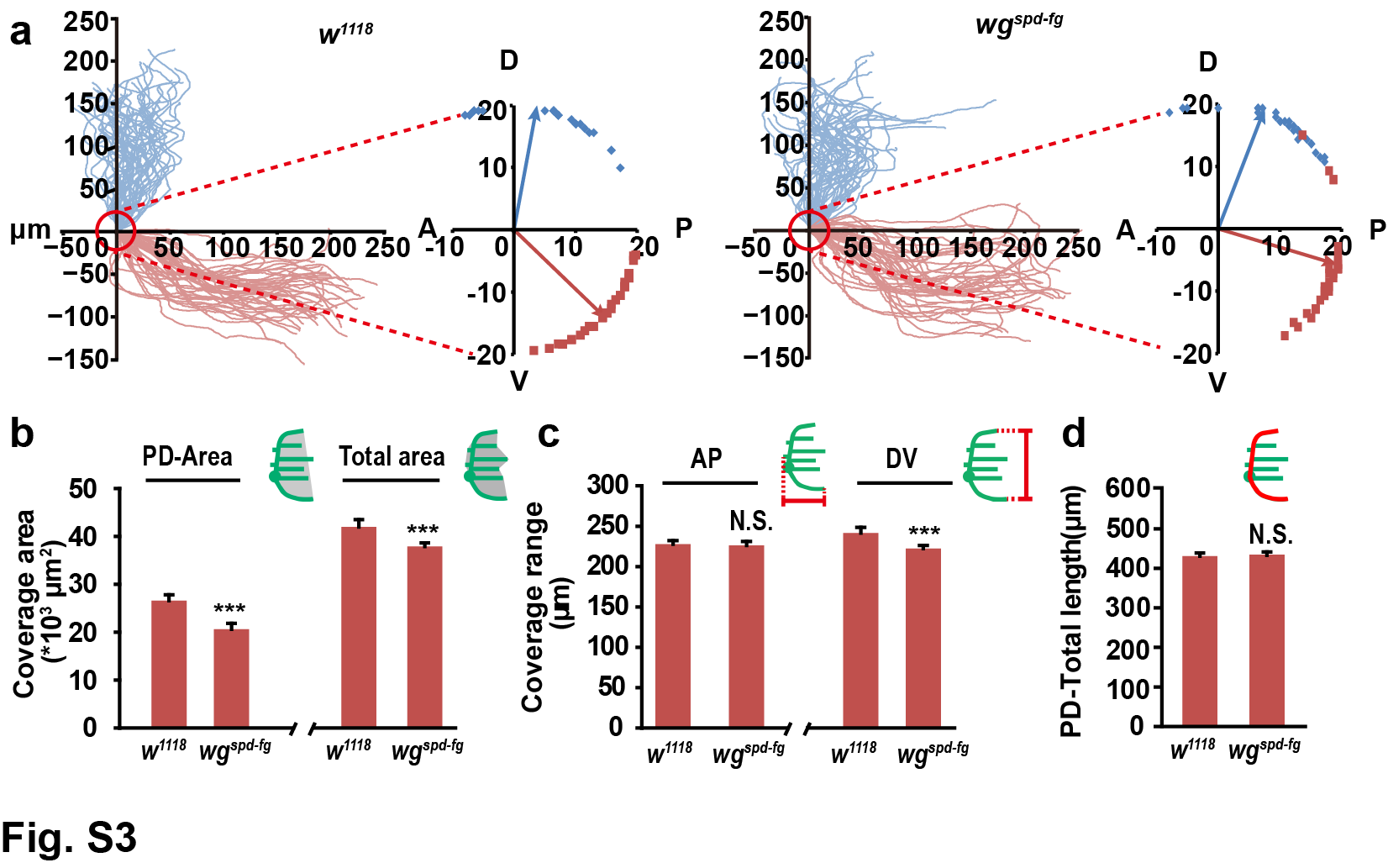

Supplement: Additional file 3: Fig S3. — Dendrite coverage area and coverage range at DV direction of ddaE neuron are statistically decreased in wg spd-fg mutants. a, The initial 20 μm parts of primary dendrites shift towards AP axis in wg spd-fg mutants. The data are obtained and presented in the same way as in Fig. 1c. b-d , The PD-Area or Total area (b), and the dendritic coverage range at DV direction (c) are significantly decreased in wgspd-fg mutants, while the PD-Total length (d) and the dendritic coverage range at AP direction (c) are unchanged. The insert cartoons show dendrites in green lines and the parameter quantified in corresponding panels. (n =42-43). (TIF 399 kb) [file 13041_2016_228_MOESM3_ESM.tif]

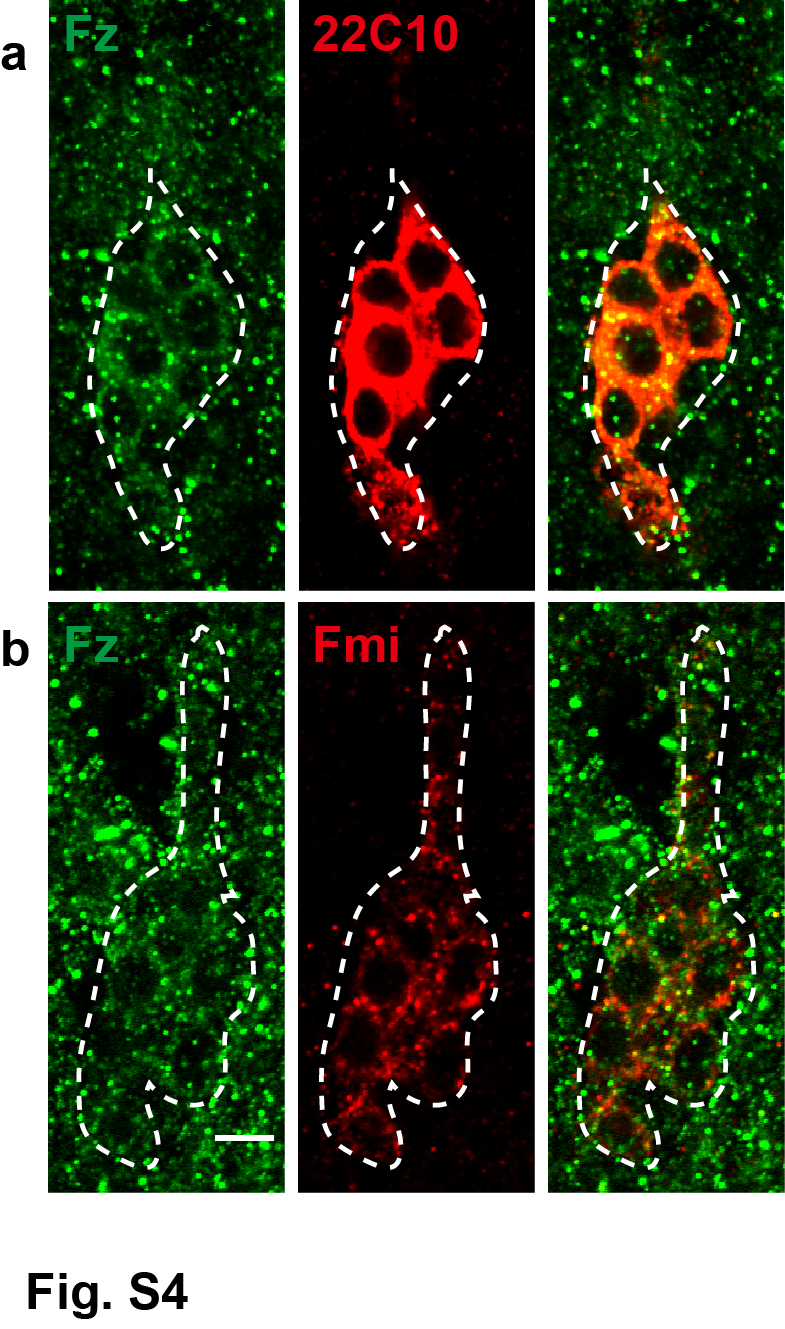

Supplement: Additional file 4: Fig S4. — Fz was expressed in ddaE and the expression level decreased in fz EY03114 mutant. a, Fz expression is detected in ddaE neuron in wild type S14 embryos. Fz protein is shown by anti-Fz antibody (green) and neurons are shown by antibody 22C10 (red). b, Fz is co-expressed with Fmi in dorsal cluster da neurons. Fz protein is shown by anti-Fz antibody (green) and neurons are shown by anti-Fmi antibody (red). Scale bar: 5 μm. (TIF 1.11 mb) [file 13041_2016_228_MOESM4_ESM.tif]

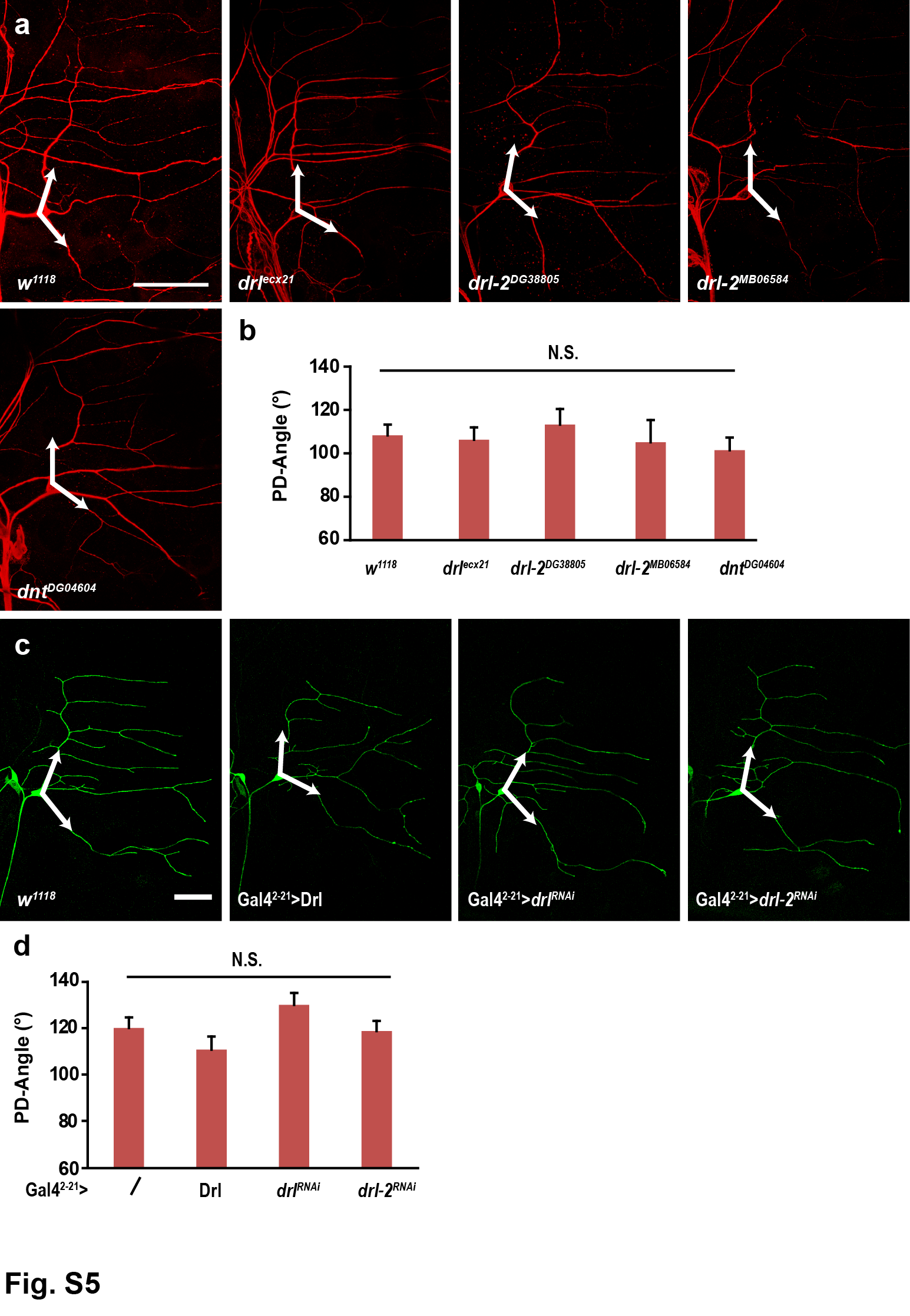

Supplement: Additional file 5: Fig S5. — The PD-angle is not affected in RYK-family receptor drl, drl-2, or dnt mutant or knock-down flies. a-b, No significant change of PD-Angle is observed in drl, drl-2, or dnt mutants. c-d, Neuronal overexpression or knock-down of either drl or drl-2 has no effect on the PD-Angle. White arrows indicate the initial parts of primary dendrites. n ≥ 30 in each group. Scale bar, 50 μm. (TIF 1.44 mb) [file 13041_2016_228_MOESM5_ESM.tif]

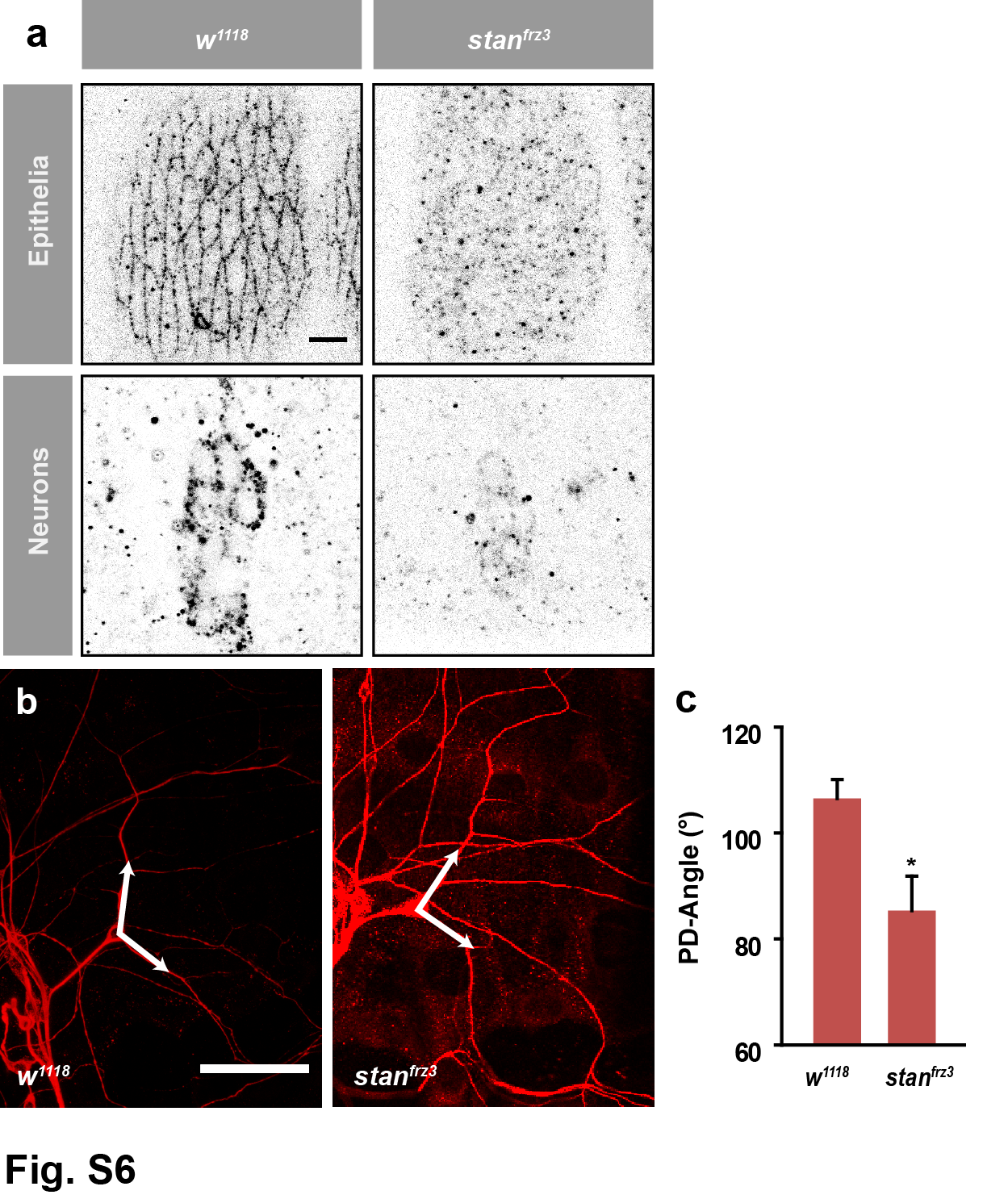

Supplement: Additional file 6: Fig S6. — Flamingo is required in ddaE neuron for dendrite directional growth. a, In homozygous available stan frz3 mutant, Fmi expression is decreased in epithelia and da neurons. b-c , The PD-Angle is significantly decreased in stan frz3 mutant. White arrows indicate the initial parts of primary dendrites. n ≥ 30 in each group. Scale bar, 5 μm in (a) and 50 μm in (b). (TIF 1.05 mb) [file 13041_2016_228_MOESM6_ESM.tif]

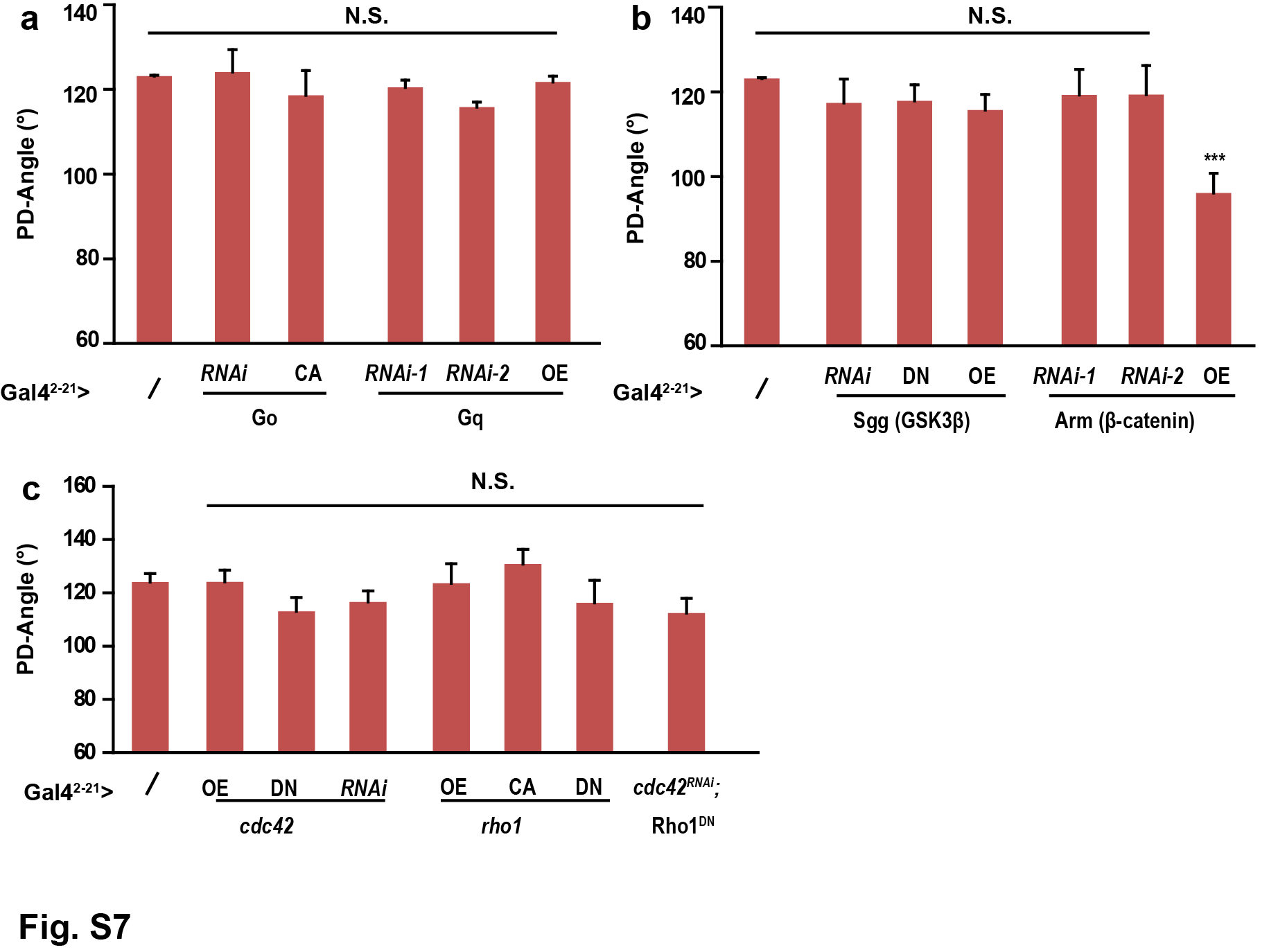

Supplement: Additional file 7: Fig S7. — Canonical-Wnt pathway and small GTPase Cdc42, Rho1 were unaffected in dendrite directional growth. a, Neither knockdown nor overexpression of Go or Gq in ddaE neurons has an effect on the PD-Angle. b, Neither knockdown nor overexpression of Sgg or Arm in ddaE neurons has an effect on the PD-Angle, except that overexpressing Arm results in a significant decrease. c, No significant change of the PD-Angle is shown when manipulating Cdc42 or/and Rho1 in ddaE neuron. CA, constitutively active form; OE, overexpression; DN, dominant negative form. n ≥ 30 in each group. (TIF 349 kb) [file 13041_2016_228_MOESM7_ESM.tif]
